# Supplementary material for: Causes and implications of the unforeseen 2016 extreme yield loss in the breadbasket of France
Source: Nat Commun. 2018 Apr 24;9:1627. doi: 10.1038/s41467-018-04087-x (PMC5915531; doi:10.1038/s41467-018-04087-x)
Supplement: Supplementary file 1 — Supplementary Information [file 41467_2018_4087_MOESM1_ESM.pdf]

Causes and implications of the unforeseen 2016 extreme yield loss in the breadbasket of France

Tamara Ben-Ari[1*], Julien Boé[2], Philippe Ciais[3], Remi Lecerf[4], Marijn Van der Velde[4] & David Makowski[1]
* Corresponding author

[1] INRA, AgroParisTech, UMR 211 Agronomie, Université Paris-Saclay, France
[2] CECI, Université de Toulouse, CERFACS/CNRS, Toulouse, France
[3] Laboratoire des Sciences du Climat et de l'Environnement, 91191 Gif-sur-Yvette, France
[4] European Commission, Joint Research Centre (JRC), Via E. Fermi 2749, I-21027 Ispra (VA), Italy

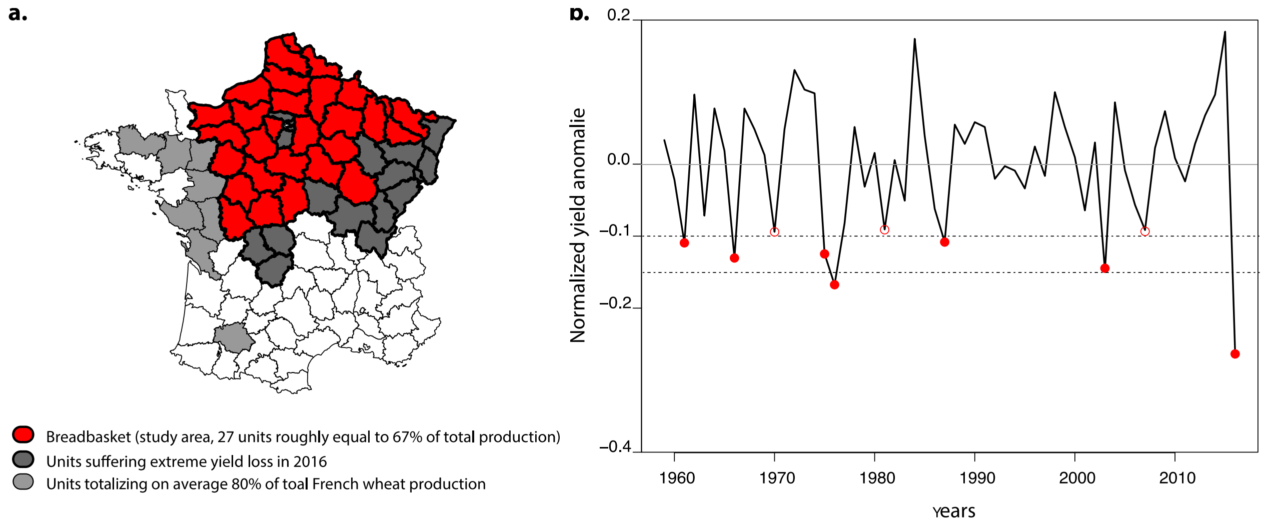

Supplementary Figure 1. Study area and yield anomalies since 1959.

a. Map of the studied areas. Over the 95 counties composing metropolitan France we focus on the so-called breadbasket (in red). In an out-of-sample procedure we also estimate yield loss probabilities in two larger regions. First, over an area composed of 35 units producing at least 80% of French wheat throughout the study period (light grey) and second in the 45 units that where the most affected in 2016 (i.e., each unit lost at least 15% of yields compared to expectation; dark grey). The map was generated with R based on the yield data used in the analyses.

b. Time series of the inter-unit median of wheat yield anomalies (for harvest years between 1959-2016) in the study area (27 units composing the breadbasket, in red in Supplementary Figure 1). Dotted lines indicate severe and extreme levels of yield losses as defined in the main (i.e., -10% and -15% yield loss). The red dots indicate yield loss below -10%. These correspond to 1961, 1966, 1975, 1976, 1987, 2003 and 2016. As an

indication, red circles indicate yield losses very close to -10% (here strictly below -9%) corresponding to 1970, 1981 and 2007. These years are respectively highlighted in dark and light grey in Figure 3.

Supplementary Figure 2

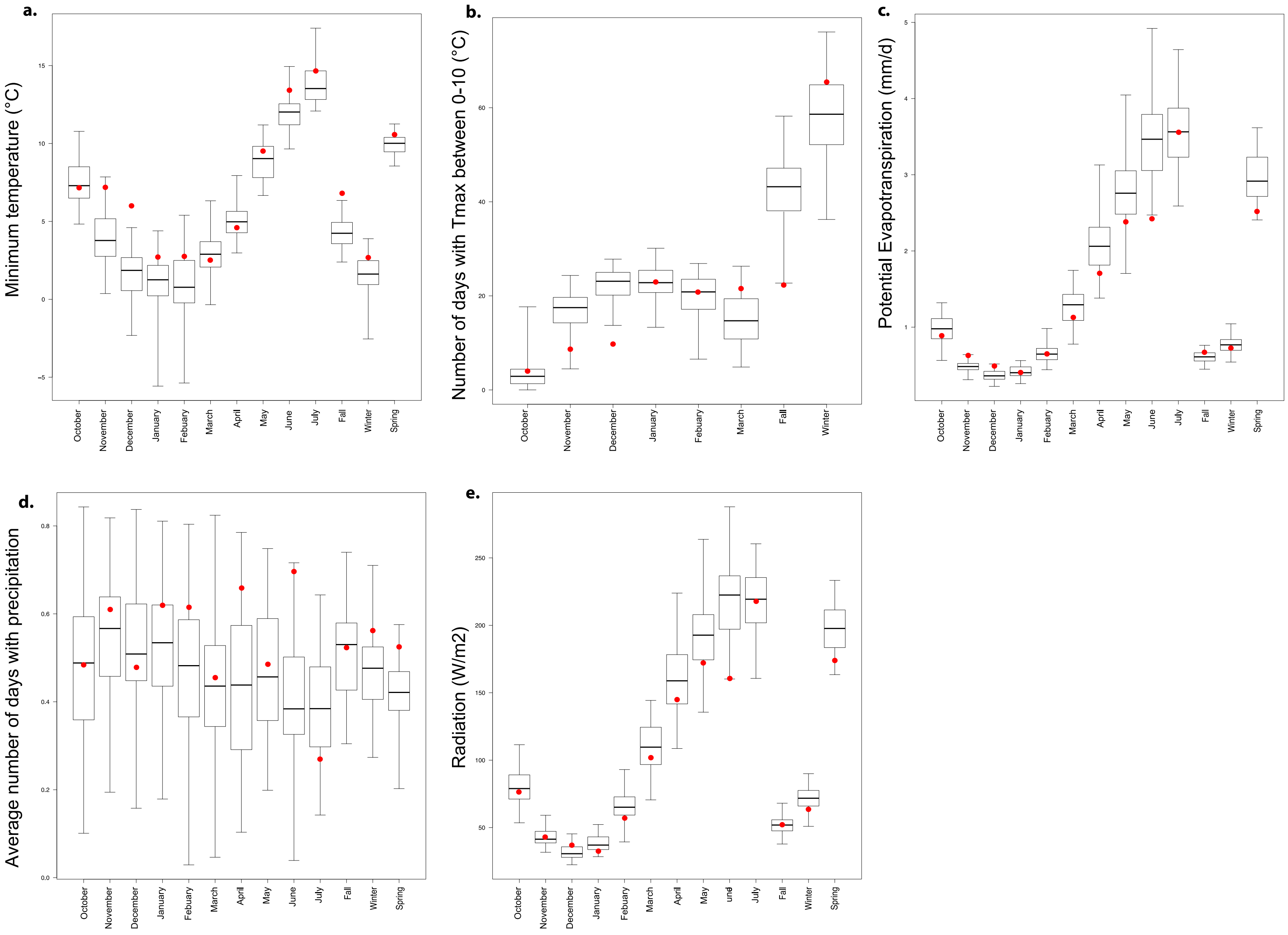

Supplementary Figure 2. Climate variable in 2016 compared to 1959-2015
Boxplot of average for (a) the number of days between 0 and 10°C, (b) potential evapotranspiration, (c) minimum temperature, (d) radiation and (e) the number of rainy days, each year over the study area for the period corresponding to the harvest years between 1959 and 2015. Whiskers extend to maximum and minimum values. Values corresponding to the 2016 harvest are presented as red dots.

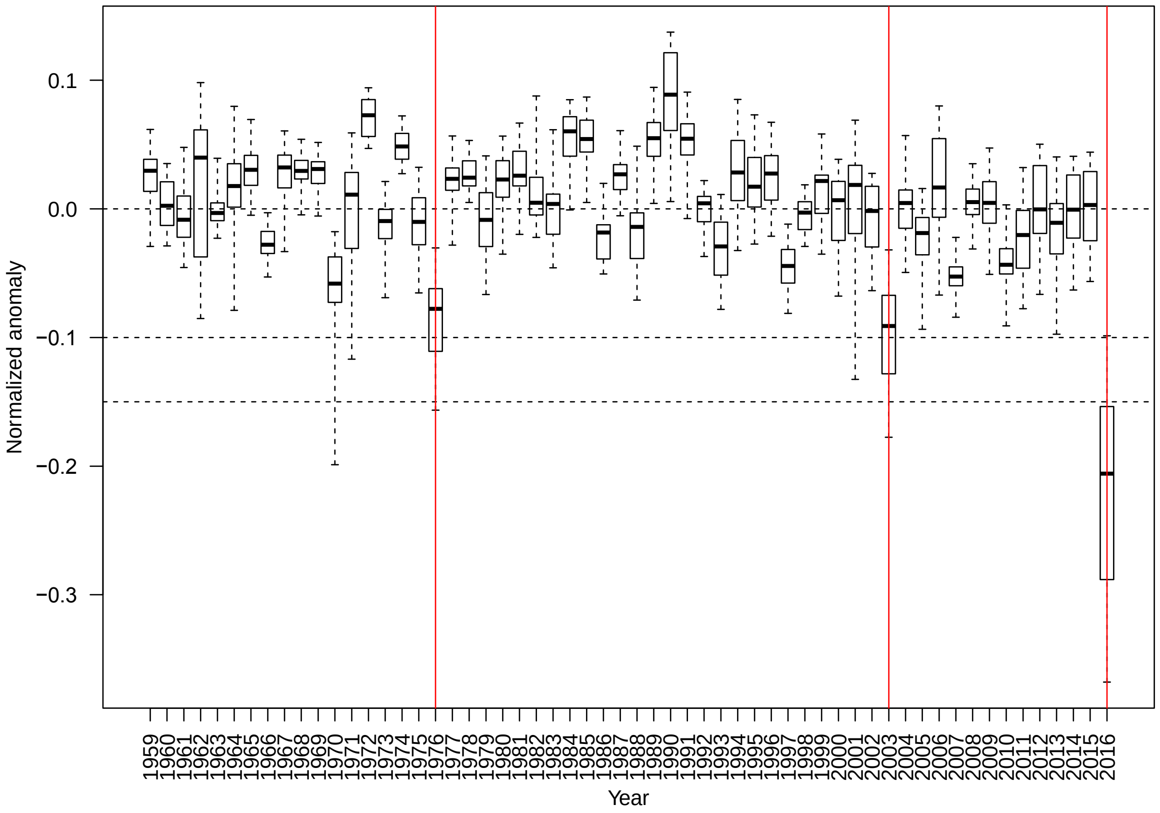

Supplementary Figure 3. Modelled normalized anomalies
Fitted normalized anomalies for the best model selected on the same set of climate variables as for the binomial logistic regressions (Supplementary Table 2). For a detailed presentation of the model see the Supplementary discussion section. Dotted horizontal grey lines indicate the three loss levels considered (below 0, -10 and -15%). Vertical red lines indicate the three major yield loss events in 1976, 2033 and 2016.

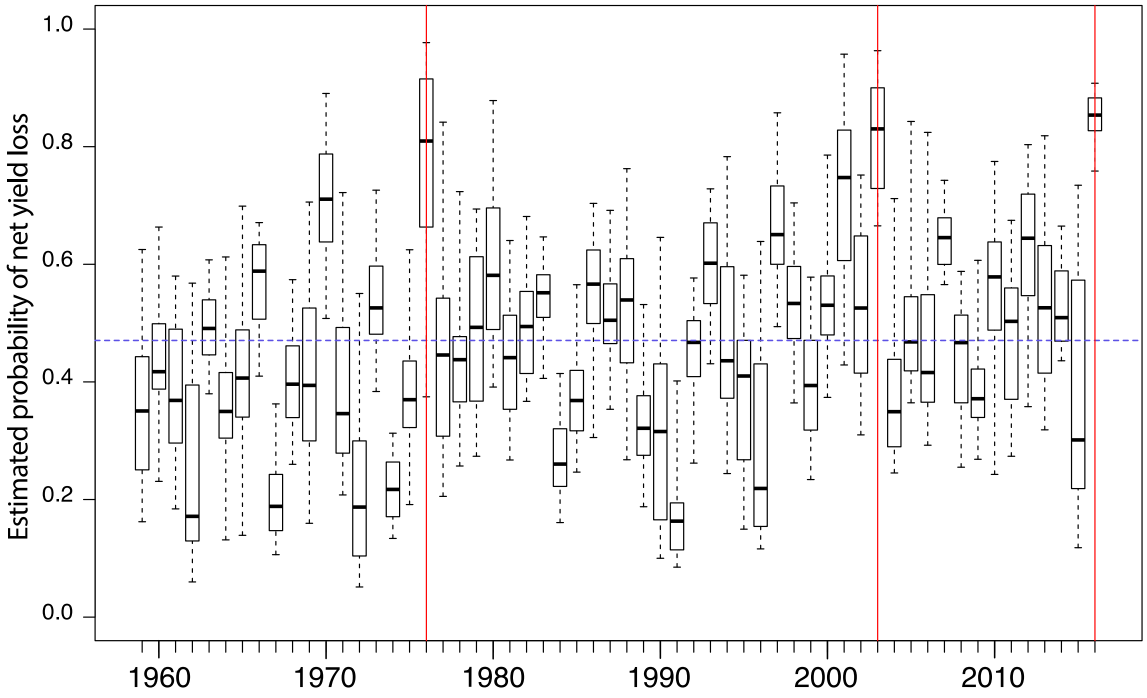

Supplementary Figure 4. Fitted probabilities of net yield loss from selected model. Boxplot of the time series of net yield loss probabilities estimated from selected statistical model presented in Supplementary Table 2 trained over the totality of the dataset in the study area. The range of the boxplot corresponds to inter-unit values of fitted probabilities in each harvested year. Red vertical line corresponds to the most extreme yield loss in 1976, 2003 and 2016. Dotted horizontal blue line corresponds to prior probability. Whiskers extend to minimum and maximum values. Note that the results for severe and extreme losses are presented in Figure 3.

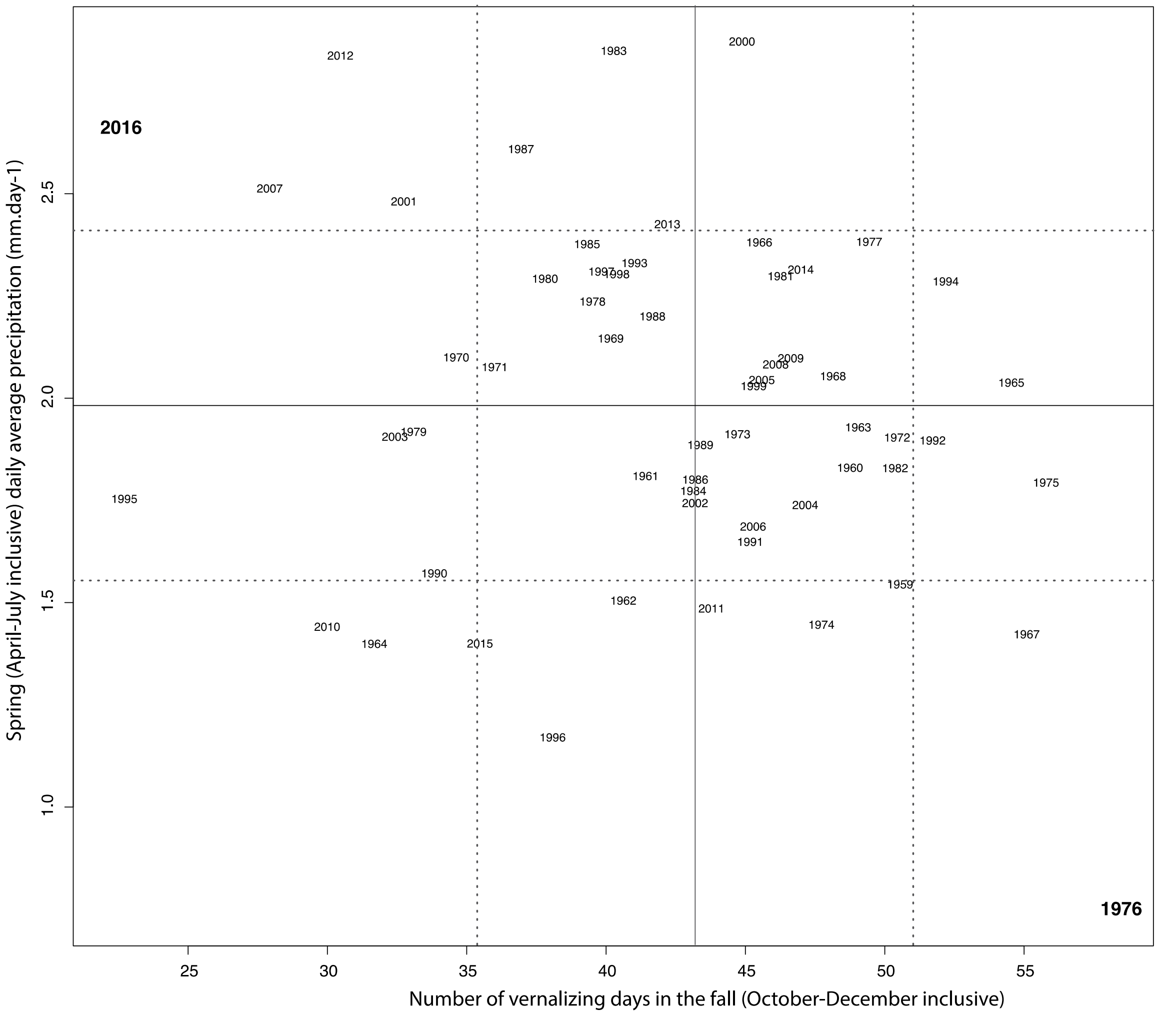

Supplementary Figure 5. Temperature in the autumn versus precipitation in the spring

Scatter plot of all harvest years for the number of vernalizing days for October-December (autumn) versus precipitation over April-July (spring). Median values are indicated as bold lines. Dotted lines correspond to one average+/- one standard deviation. Years 2016 and 1976 are highlighted in bold.

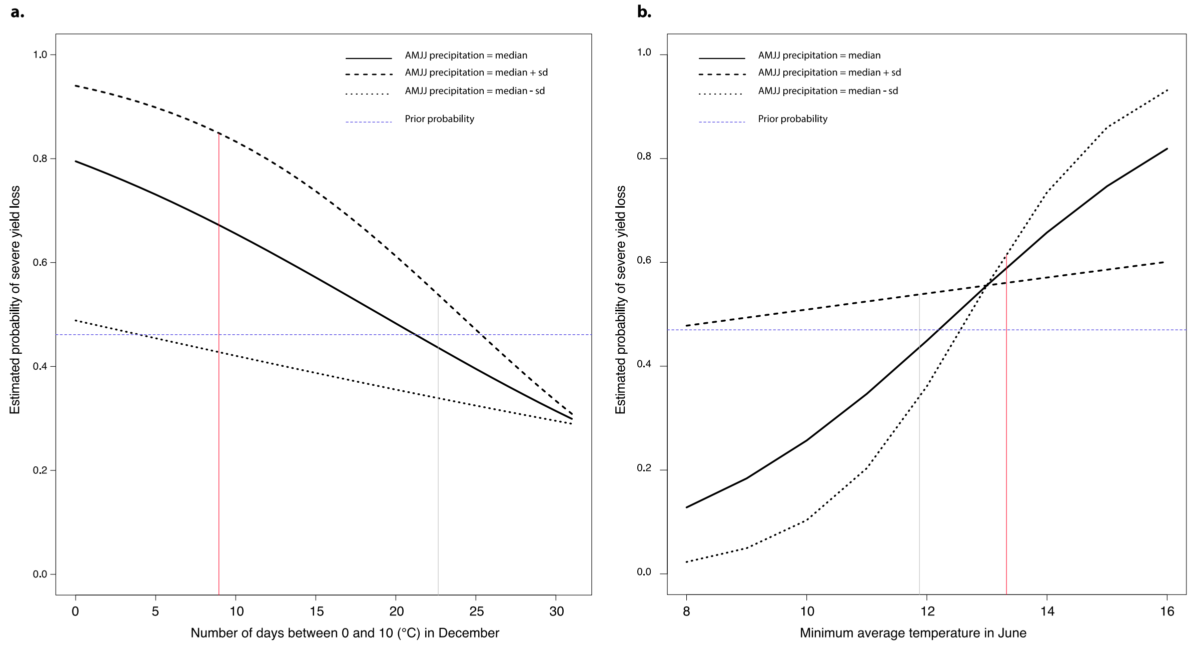

Supplementary Figure 6. Modeled temperature – precipitation interactions for severe yield losses

(a) Autumn-spring interaction

Modeled impacts of the interaction between the number of days with temperature below 10°C in December and spring precipitation on the probability of severe yield loss. Vertical grey line: median number of days with Tmax between 0 and 10°C in the dataset; red line: values in 2016. Horizontal dotted blue lines correspond to prior probabilities. These relationships are presented as a function of spring precipitation for three situations average daily precipitation is equal to its median (bold line) or equal to +/- one standard deviation (dotted lines).

(b) spring-spring interaction

Modeled impacts of minimum temperatures in June and precipitation in the spring, on the probability of severe yield loss. Vertical grey line: median June Tmin in the dataset; red line: values in 2016. Horizontal dotted blue lines correspond to prior probabilities. These relationships are presented as a function of spring precipitation for three situations average daily precipitation is equal to its median (bold line) or equal to +/- one standard deviation (dotted lines).

Figure S7

Probability of net yield loss

Breadbasket - 27 departements

35 departements

45 departements

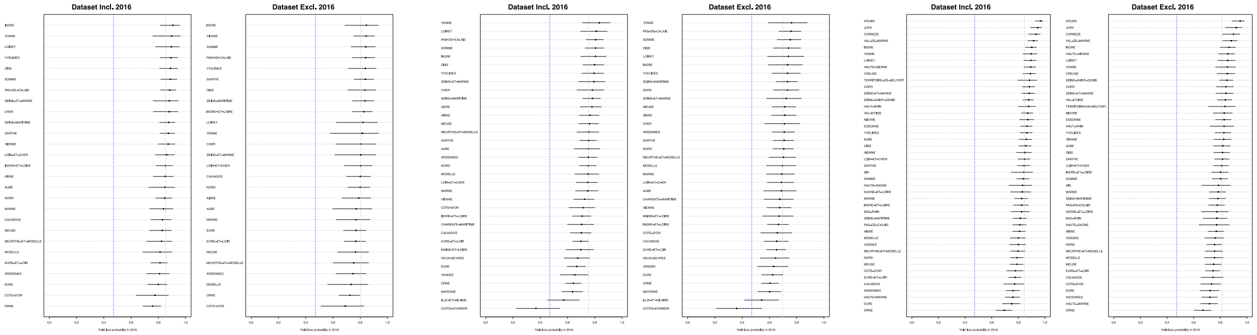

Probability of severe yield loss

Breadbasket - 27 departements

35 departements

45 departements

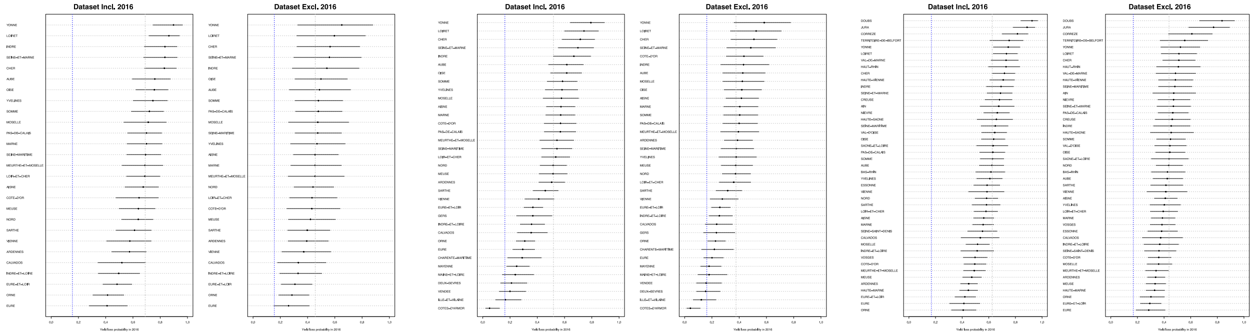

Probability of extreme yield loss

Breadbasket - 27 departements

35 departements

45 departements

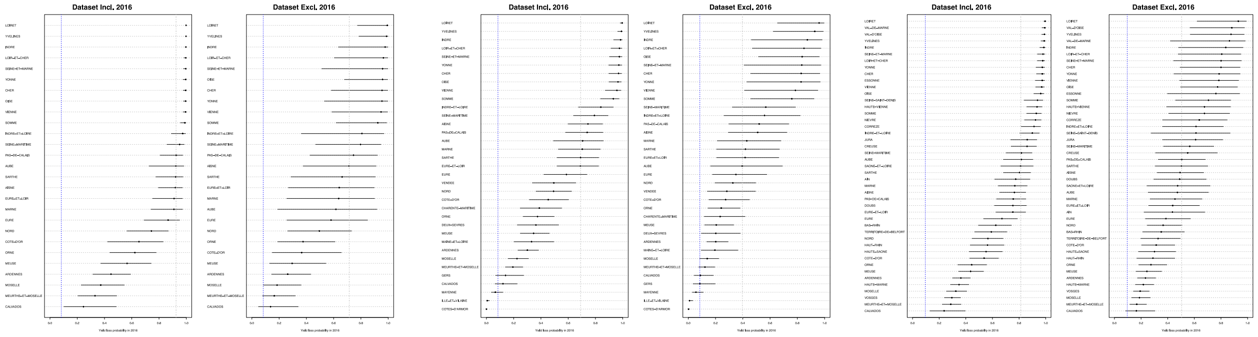

Supplementary Figure 7. Estimated probabilities and confidence intervals in each unit of the 'breasbasket' and in two larger areas.

Probabilities of net, severe and extreme yield loss from the best model trained in the study area from the dataset excluding 2016 in an out-of-sample procedure. Best models are described in Supplementary Table 2; confidence intervals are indicated as black segments. Blue dotted line indicates prior probabilities (as defined in Supplementary Table 3). Median estimated probabilities over all administrative units are indicated by dotted dark grey line. A similar figure is successively presented for the three levels of yield losses (a-c) the study area, (d-f) in an area producing 80% of total wheat in France and composed of 35 counties and (g-i) an area composed of 45 counties that where the most affected by 2016 extreme yield loss event (see also Supplementary Figure 1a. for a description of the two additional areas). Probabilities on these graphs must be considered as distances to prior probabilities.

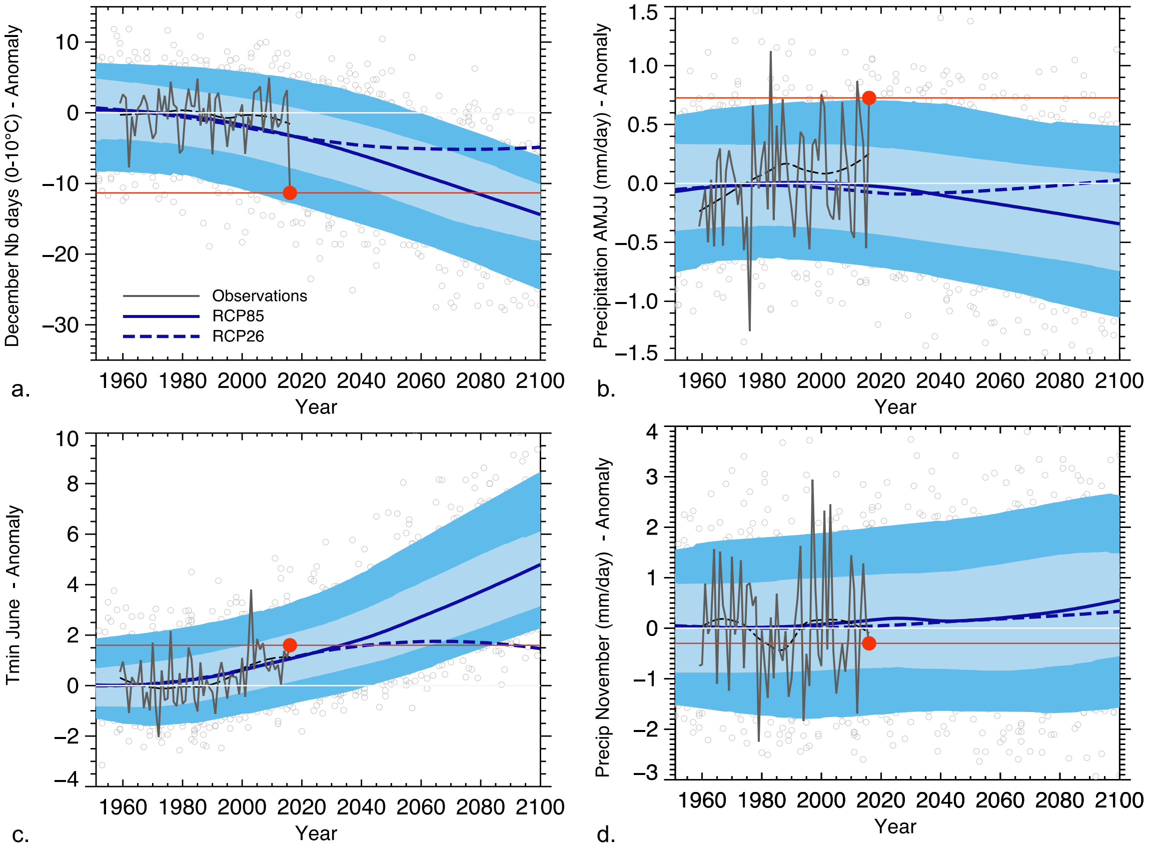

Supplementary Figure 8. Time evolution of the four climate indices with a significant observed association with wheat yield loss (RCP 8.5). The anomalies relative to the 1959-1988 reference period are shown (see methods) for **(a)** Number of days between 0 and 10°C, **(b)** Precipitation in spring (AMJJ, mm per day), **(c)** Minimum temperature in June (°C) and **(d)** Precipitation in November (mm per day). The dark blue lines show the multi-model median derived from an ensemble of 13 CMIP5 models and estimated by locally weighted scatterplot smoothing (LOESS) (solid line: RCP8.5 scenario. Dashed line: RCP2.6 scenario). SAFRAN results are shown with dark grey lines (black dashed line: LOESS filter results, thin line: interannual values). The light (dark) blue shaded areas show the 20-80% (5-95%) range of the models distribution for interannual values assuming the independence of models, for the historical and

RCP85 simulations. The associated delimiting quantiles are estimated with a local polynomial quantile regression. The gray points show the interannual values for the models.

Supplementary Table 1

Description of post-harvest diagnoses of the impact of various climatic conditions during the 2015-2016 soft wheat-growing season on different phenological stages based on technical literature, extension services or expertise magazines.

Factors	Time period	Months	Spatial extent	Mechanism involved	References
**Excess precipitation**	Late Spring	May-June	East, North and central France	Root asphyxiation	(1,2)
**Favorable temperature and Radiation**	Early Spring	March-April	Not specified	Favorable to biomass formation but with fragility to lodging	(2)
**Excess precipitation and lack of radiation**	Spring	May-June	East, North and central parts of the France	Promoted septoriosis, yellow or red rust, fusarium head blights, microdochium spreads<br><br>Florets abortion, ovule fertilization	(1,2,3)
**Above average temperatures**	Autumn/ Winter	October to February	France	Created favorable conditions to both virus spreading and rust infectious potential<br>Favors the development of weeds<br>Advanced development stage<br>Aphids pressure<br>Rust inoculum	(2,4)
**High temperatures**	Winter	Not specified	France	High Biomass<br>Early growth stages<br>Rust inoculum	(3)
**Low temperatures**	Winter	Feb-March	France	Growth slowdown<br>Reduction in diseases<br>Lodging risks increase	(3)

Supplementary Table 2

Symbol used

Some of the figures presented below use the a code for variable names:
Tx stands for Tmax and *Tn* for Tmin, *Tx_0_10* stands for the number of days with Tmax between 0 and 10°C, *rv* for radiation, *etp* for potential evapotranspiration, *pr* for precipitation and pr_per for the average number of days with rain per month. A suffix indicates the period in the growing season: *O* for October, *N* for November, *D* for December, *Ja* for January, *F* for February, *Ma* for March, *A* for April, *My* for May, *Ju* for June and *Jy* for July. OND corresponds to October-December (autumn) and AMJJ to April-July (spring).

a. Initial selection

List of all variables tested in the statistical model. Each month and season is tested for October-December and April-July and for each yield loss intensity (net, severe, extreme). Wee look for the best temperature and precipitation/humidity variables, non correlated or redundant and based on the BIC criteria we compute one statistical model per yield loss level.

YIELD LOSS				SEVERE YIELD LOSS				EXTREME YIELD LOSS			
Best Temperature and Precip variable for each univariate model				Best Temperature and Precip variable for each univariate model				Best Temperature and Precip variable for each univ			
FALL		SPRING		FALL		SPRING		FALL		SPRING	
Name	BIC	Name	BIC	Name	BIC	Name	BIC	Name	BIC	Name	BIC
tx_0_10_D	2150.960182	tn_Ju	2070.208153	pr_per__N	1312.88958	tn_Ju	1268.357698	pr_per__N	868.966791	tn_Ju	812.510427
pr_N	2159.642921	tx34_Ju	2132.301563	pr_N	1328.26016	tx34_Ju	1318.289098	tx_0_10_D	873.819346	tx34_Ju	874.789898
pr_D	2162.172655	tn_AMJJ	2142.094789	tx_0_10_D	1335.73298	tx34_AMJJ	1340.041169	tn_D	884.869939	tx34_AMJJ	884.574526
pr_per__N	2164.00573	pr_AMJJ	2142.442967	tn_D	1345.7641	tx_Ju	1346.647649	pr_N	887.139882	tx_Ju	891.749808
pr_OND	2165.271055	tx34_AMJJ	2144.310555	tx_O	1350.78337	tn_AMJJ	1349.834369	tx_D	887.782674	pr_My	892.806092
pr_per__D	2165.662098	pr_Ju	2151.271752	tx_D	1350.90889	pr_AMJJ	1350.379914	tn_N	888.877025	tn_AMJJ	893.540603
etp_D	2168.666113	tx_Ju	2154.316459	rv_N	1351.8126	rv_AMJJ	1357.334709	tx_0_10_ON	891.585016	pr_per__A	897.701689
tx_0_10_OND	2169.752541	pr_Jy	2162.507837	tn_O	1351.96174	pr_My	1357.393048	tx_N	894.078864	rv_Ju	898.547378
rv_N	2170.228575	pr_per__AMJJ	2163.36433	tn_N	1352.23738	pr_Ju	1358.011352	tn_O	895.458163	rv_AMJJ	898.725529
rv_D	2170.563625	tn_Jy	2165.60045	tx_0_10_ON	1359.09592	etp_AMJJ	1358.08174	tx_0_10_N	895.90393	pr_AMJJ	898.908792
pr_per__OND	2171.973642	pr_per__A	2165.697185	tx_0_10_N	1359.43291	pr_per__A	1358.5141	tx_O	899.548601	etp_AMJJ	898.940009
tx34_OND	2172.885283	tx34_Jy	2166.010484	pr_OND	1359.46757	etp_My	1360.701047	tn_OND	899.798539	pr_Ju	899.829875
tx34_O	2172.885283	pr_per__Ju	2172.680159	tx_N	1359.52104	pr_per__AMJJ	1361.24726	rv_N	900.193687	pr_per__Ju	901.521883
tx34_OND	2172.885283	tx34_A	2172.885283	pr_per__ON	1359.75128	rv_My	1361.837739	tx34_OND	903.326591	etp_My	902.090809
tn17_O	2172.885283	tn17_A	2172.885283	etp_D	1361.02688	etp_Jy	1361.960837	tx34_O	903.326591	tx34_A	903.326591
tx34_N	2172.885283	tn17_AMJJ	2172.885283	etp_O	1361.06562	rv_Ju	1362.420656	tx34_OND	903.326591	tn17_A	903.326591
tn17_N	2172.885283	tn17_My	2172.885283	tn_OND	1361.76695	tx34_A	1362.440461	tn17_O	903.326591	tn17_AMJJ	903.326591
tx34_D	2172.885283	tn17_Ju	2172.885283	tx_0_10_O	1361.90451	tn17_A	1362.440461	tx34_N	903.326591	tn17_My	903.326591
tx_0_10_N	2172.955651	tn17_Jy	2172.885283	tx34_OND	1362.44046	tn17_AMJJ	1362.440461	tn17_N	903.326591	tn17_Ju	903.326591
tn17_D	2175.143722	tx_AMJJ	2174.357044	tx34_O	1362.44046	tn17_My	1362.440461	tx34_D	903.326591	tn17_Jy	903.326591
tn17_OND	2175.143722	pr_My	2174.944127	tx34_OND	1362.44046	tn17_Ju	1362.440461	tx_OND	903.335726	tx34_Jy	903.94173
tn_N	2175.170215	pr_per__Jy	2175.612519	tn17_O	1362.44046	tn17_Jy	1362.440461	etp_D	904.463158	rv_My	905.250402
tx_O	2175.285908	tx_0_10_My	2175.615834	tx34_N	1362.44046	tn_A	1364.09259	pr_per__O	905.698146	etp_A	905.588592
tn_O	2175.656028	tx_0_10_Ju	2176.113667	tn17_N	1362.44046	tx34_Jy	1364.127759	etp_N	906.345538	etp_Ju	905.729225
etp_N	2175.945279	tn_A	2176.452559	tx34_D	1362.44046	etp_A	1364.192786	etp_O	906.745574	etp_Jy	905.823638
tn_D	2176.546789	etp_A	2176.937663	tn17_D	1367.07911	pr_per__Ju	1364.274203	pr_per__ON	908.156137	tx_0_10_My	905.861071
tx_0_10_O	2177.619281	tx34_My	2177.223923	tn17_OND	1367.07911	rv_A	1365.353088	pr_O	908.301354	pr_per__AM	906.139537
pr_per__O	2178.010674	tx_0_10_A	2177.451667	tx_OND	1367.17488	tx_0_10_My	1366.08271	tx_0_10_O	908.577224	tn_A	906.519698
etp_OND	2178.36859	rv_A	2177.598288	etp_N	1367.42507	rv_Jy	1366.289302	pr_D	908.674526	pr_per__My	906.749302
pr_O	2178.574741	rv_Jy	2177.649359	rv_OND	1367.80252	tn_My	1367.117994	rv_O	908.777182	rv_A	906.818407
rv_OND	2178.641003	rv_AMJJ	2178.230742	rv_D	1368.06869	tx_My	1367.240544	tn17_D	909.292551	tn_My	907.485838
tn_OND	2178.807706	rv_Ju	2178.378976	pr_per__O	1368.13519	etp_Ju	1367.581902	tn17_OND	909.292551	tx_0_10_Ju	907.792724
rv_O	2179.023848	etp_Jy	2178.7574	pr_per__D	1369.00797	pr_Jy	1367.894517	pr_OND	910.028786	rv_Jy	908.496106
tx_D	2179.3881	pr_A	2179.26086	rv_O	1369.39689	tn_Jy	1367.970605	pr_per__D	910.551241	tx_My	908.652703
tx_OND	2180.012943	tx_My	2179.413862	etp_OND	1369.492	tx_AMJJ	1368.194813	rv_OND	910.605413	tx_AMJJ	909.272228
etp_O	2180.058098	etp_My	2179.498325	pr_D	1369.74122	tx_0_10_Ju	1368.746031	rv_D	910.616649	pr_per__Jy	909.62193
tx_N	2180.190247	tx_0_10_AMJJ	2179.619854	pr_O	1369.77847	tx_A	1368.870959	etp_OND	910.667392	tn_Jy	909.693231
		tx_Jy	2179.699485			tx_0_10_A	1368.884744			tx_A	910.186145
		rv_My	2179.702013			pr_A	1369.010181			tx34_My	910.335992
		pr_per__My	2179.810947			tx34_My	1369.118759			tx_0_10_AM	910.442333
		etp_AMJJ	2179.939554			pr_per__My	1369.310156			pr_Jy	910.515243
		tn_My	2179.940421			pr_per__Jy	1369.547421			tx_0_10_A	910.609388
		tx_A	2180.066077			tx_Jy	1369.641994			tx_Jy	910.658059
		etp_Ju	2180.128737			tx_0_10_AMJJ	1369.76345			tx_0_10_Jy	910.661755
		tx_0_10_Jy	2180.241096			tx_0_10_Jy	1369.787462			pr_A	910.68147

b-d. Model summary for each yield loss level considered

These variables are then combined with and without interaction and selected from a stepwise selection process. We present the summary of each of these models in one tab per yield loss intensity. The significance of each covariate and the parameter estimation are presented in each table. The Bayesian Information Criteria is used to select the most parsimonious model. Area Under the Curve (AUC) and ROC analysis score of prediction accuracy

b.

Net Yield loss - Best model summary

```
tx_0_10_D       X1
pr_N            X2
tn_Ju           X5
pr_AMJJ         X6

Call:
glm(formula = Anomaly ~ X1 + X2 + X5 + X6 + X1:X6 + X5:X6, family = "binomial")

Deviance Residuals:
Min      1Q   Median     3Q     Max
-1.9449  -1.0342  -0.5513   1.1064   2.1810

Coefficients:
Estimate Std. Error z value Pr(>|z|)
(Intercept) -25.36265    2.92769   -8.663  < 2e-16 ***
X1          0.09077    0.05680    1.598  0.11005
X2          0.13861    0.04830    2.870  0.00411  **
X5          1.78528    0.22330    7.995 1.29e-15 ***
X6         10.82755    1.48063    7.313 2.62e-13 ***
X1:X6      -0.08231    0.02747   -2.996  0.00274  **
X5:X6      -0.68960    0.10904   -6.324 2.55e-10 ***
---
Signif. codes:  0 ‘***’ 0.001  ‘**’ 0.01 ‘*’ 0.05 ‘.’ 0.1 ‘ ’ 1

(Dispersion parameter for binomial family taken to be 1)

Null deviance: 2165.5  on 1565   degrees of freedom
Residual deviance: 1932.8  on 1559   degrees of freedom
AIC: 1946.8

Number of Fisher Scoring iterations: 4
```

**BIC**	**1984.302**
**AUC**	**0.702121**

c.

Severe Yield loss - Best model summary

pr_per__N	X1
tx_0_10_D	X2
tn_Ju	X5
pr_AMJJ	X6

Call:
glm(formula = Anomaly ~ X1 + X2 + X5 + X6 + X1:X6 + X2:X6 + X5:X6, family = "binomial")

Deviance Residuals:

Min	1Q	Median	3Q	Max
-1.8987	-0.5875	-0.4198	-0.2347	2.9980

Coefficients:

	Estimate	Std. Error	z value	Pr(>|z|)	
(Intercept)	-27.71059	3.55653	-7.791	6.62e-15	***
X1	8.96694	2.15467	4.162	3.16e-05	***
X2	0.23333	0.07071	3.300	0.000968	***
X5	1.16851	0.24981	4.678	2.90e-06	***
X6	10.09938	1.72675	5.849	4.95e-09	***
X1:X6	-3.03888	0.99918	-3.041	0.002355	**
X2:X6	-0.14971	0.03161	-4.736	2.18e-06	***
X5:X6	-0.36101	0.12074	-2.990	0.002789	**

Signif. codes: 0 ‘***’ 0.001 ‘**’ 0.01 ‘*’ 0.05 ‘.’ 0.1 ‘ ’ 1

(Dispersion parameter for binomial family taken to be 1)

Null deviance: 1355.1 on 1565 degrees of freedom
Residual deviance: 1136.6 on 1558 degrees of freedom
AIC: 1152.6

**BIC**	**1195.402**
**AUC**	**0.7550625**

d.

Extreme Yield loss - Best model summary

pr_per__N	X1
tx_0_10_D	X2
tn_Ju	X5
pr_My	X6

```
Call:
glm(formula = Anomaly ~ X1 + X2 + X5 + X6 + X2:X6 + X5:X6, family = "binomial")

Deviance Residuals:
Min       1Q   Median       3Q      Max
-1.7398  -0.3766  -0.2537  -0.1681   3.0763

Coefficients:
Estimate Std. Error z value Pr(>|z|)
(Intercept) -28.00648    3.25343   -8.608   < 2e-16 ***
X1          4.05692    0.75917    5.344  9.10e-08 ***
X2          0.41742    0.06825    6.116  9.60e-10 ***
X5          1.06084    0.22266    4.764  1.90e-06 ***
X6          8.02909    1.35872    5.909  3.44e-09 ***
X2:X6       -0.20031    0.02595   -7.719  1.18e-14 ***
X5:X6       -0.27408    0.09811   -2.794   0.00521  **
---
Signif. codes:  0 '***' 0.001 '**' 0.01 '*' 0.05 '.' 0.1 ' ' 1

(Dispersion parameter for binomial family taken to be 1)

Null deviance: 895.97   on 1565   degrees of freedom
Residual deviance: 648.71   on 1559   degrees of freedom
AIC: 662.71

Number of Fisher Scoring iterations: 6
```

**BIC**	**700.2024**
**AUC**	**0.8291997**

Supplementary Table 3

Odds and risk ratios of net, severe and extreme yield loss in the French Breadbasket in 2016 computed from prior and estimated probabilities from a dataset trained in the study area from 1958 to 2016 (dataset including the 2015-2016 growing season) and from 1958 to 2015 (dataset excluding the 2015-2016 growing season). Information added by the statistical model can be viewed as the difference between prior and posterior probabilities. Posterior probabilities are presented by their median and 10-90th percentiles probability values. The full inter-*departements* distribution and estimated probabilities and their confidence intervals are presented in Supplementary Figure 7.

**NET YIELD LOSS<br>Median values accross counties**				
	Prior	Posterior Probability	**Risk ratio**	**Odds ratio**
Training dataset Excl. 2016	0.46	0.80 (0.74-0.84)	1.74	4.80
Training dataset Incl. 2016	0.47	0.85 (0.81-0.89)	1.81	6.57

**SEVERE YIELD LOSS<br>Median values accross counties**				
	Prior	Posterior Probability	**Risk ratio**	**Odds ratio**
Training dataset Excl. 2016	0.14	0.46 (0.32-0.56)	3.23	5.11
Training dataset Incl. 2016	0.16	0.69 (0.49-0.84)	4.42	12.03

**EXTREME YIELD LOSS<br>Median values accross counties**				
	Prior	Posterior Probability	**Risk ratio**	**Odds ratio**
Training dataset Excl. 2016	0.07	0.71 (0.23-0.97)	10.67	34.83
Training dataset Incl. 2016	0.08	0.93 (0.42-0.99)	11.14	136.37

Supplementary discussion

To check whether our results were robust to a change in statistical model, we fitted a series of models using the same selection procedure as described before but with normalized anomalies as the response variable.

We first fit each climatic variable independently to normalized yield anomalies – per yield loss levels – and select the best covariates based on BIC. Then we combine the selected variables using a stepwise selection based on BIC. We found that the model leading to the lowest BIC included the same variables as those of the binomial logistic model, i.e., with the number of days between 0 and 10°C in the autumn, the number of rainy days in November, minimum temperature in June and precipitation in the spring (AMJJ).

As above, two interactions are also selected: between with the number of days between 0 and 10°C and precipitation in the spring and the interaction between temperature in June and precipitation in the spring. All covariates are highly significant (p-value <

0.001). Supplementary Figure 4 shows that the predictions of normalized yield anomalies and predicted probabilities of yield loss follow similar patterns. A few additional moderate loss years were also identified by the linear model (1966,1970 and 2007).

Supplementary References

1. Semences de France (2016) Rendements catastrophiques du blé en 2016 : la pluie, seule responsable? Available at: http://www.semencesdefrance.com/actualite-semences-de-france/rendements-catastrophiques-ble-2016-pluie-seule-responsable/.
2. Académie d'agriculture de France (2016) La production de céréales à paille en France en 2016 - qualité sanitaire et technologique des grains, quelques éléments d'explication à partir de l'exemple du blé tendre.
3. Jean-Charles DESWARTE (Arvalis) (2017) Bilan agro-climatique et conséquences sur le rendement des céréales à paille.
4. FranceAgriMer (2016) CereObs. Available at: https://cereobs.franceagrimer.fr/Pages/publications.aspx [Accessed February 5, 2017].
