# Supplementary material for: Causes and implications of the unforeseen 2016 extreme yield loss in the breadbasket of France
Source: Nat Commun. 2018 Apr 24;9:1627. doi: 10.1038/s41467-018-04087-x (PMC5915531; doi:10.1038/s41467-018-04087-x)
Supplement: Supplementary file 3 — Description of Additional Supplementary Files [file 41467_2018_4087_MOESM3_ESM.pdf]

Description of Additional Supplementary Files

Supplementary Movie 1. Normalized wheat Yield anomalies in France from 1959 to 2016. Anomalies are expressed as percentage of expected values. The breadbasket, composed of 27 counties, is delineated in bold black contours. For clarity, units with anomalies between -5 and +5 % are shown in light yellow. The maps were generated with R based on the yield data used in the analyses.
